# Supplementary material for: The impact of injury of the chorda tympani nerve during primary stapes surgery or cochlear implantation on taste function, quality of life and food preferences: A study protocol for a double-blind prospective prognostic association study
Source: PLoS One. 2023 May 18;18(5):e0284571. doi: 10.1371/journal.pone.0284571 (PMC10194866; doi:10.1371/journal.pone.0284571)
Supplement: S2 File — (DOCX) [file pone.0284571.s002.docx]

- **The impact of injury of the chorda tympani nerve during primary stapes surgery or primary cochlear implantation**

**on taste function, quality of life and food preferences**

**(August 2021)**

**‘The impact of injury of the chorda tympani nerve during primary stapes surgery and primary cochlear implantation on taste function, quality of life and food preferences’**

| **Protocol ID** | NL76749.041.21 |
| --- | --- |
| **Short title** | **T**aste function **A**fter **C**h**O**rda tympani injury – The TACO study |
| **EudraCT number** | *Not applicable* |
| **Version** | 2.0 |
| **Date** | 03-08-2021 |

# TABLE OF CONTENTS

PROTOCOL SIGNATURE SHEET 4

TABLE OF CONTENTS 5

LIST OF ABBREVIATIONS AND RELEVANT DEFINITIONS 7

1. INTRODUCTION AND RATIONALE 9

2. OBJECTIVES 11

3. STUDY DESIGN 12

4. STUDY POPULATION 14

4.1 Population base 14

4.2 Inclusion criteria 14

4.3 Exclusion criteria 14

4.4 Sample size calculation 14

5. NON-INVESTIGATIONAL PRODUCT 5.1 Taste Strip Test 16

5.4 Electrogustometer 17

6. METHODS 19

6.1 Study parameters/endpoints 19

6.1.1 Main study parameter 19

6.1.2 Secondary study parameters 19

6.1.3 Other study parameters 20

6.2 Randomisation, blinding and treatment allocation 20

6.3 Study procedures 20

6.4 Withdrawal of individual subjects 21

6.4.1 Specific criteria for withdrawal 21

6.5 Replacement of individual subjects after withdrawal 21

6.6 Follow-up of subjects withdrawn from treatment 21

6.7 Premature termination of the study 21

7. SAFETY REPORTING 22

7.1 Temporary halt for reasons of subject safety 22

7.2 AEs, SAEs and SUSARs 22

7.2.1 Adverse events (AEs) 22

7.2.2 Serious adverse events (SAEs) 22

7.2.3 Suspected unexpected serious adverse reactions (SUSARs) 23

7.3 Annual safety report 23

7.4 Follow-up of adverse events 23

7.5 [Data Safety Monitoring Board (DSMB) / Safety Committee] 23

8. STATISTICAL ANALYSIS 24

8.1 Primary study parameter 24

8.2 Secondary study parameters 24

8.3 Other study parameters 24

8.4 Interim analysis 24

9. ETHICAL CONSIDERATIONS 25

9.1 Regulation statement 25

9.2 Recruitment and consent 25

9.3 Objection by minors or incapacitated subjects 25

9.4 Benefits and risks assessment, group relatedness 25

9.5 Compensation for injury 26

9.6 Incentives 26

10. ADMINISTRATIVE ASPECTS, MONITORING AND PUBLICATION 27

10.1 Handling and storage of data and documents 27

10.2 Monitoring and Quality Assurance 28

10.3 Amendments 28

10.4 Annual progress report 28

10.5 Temporary halt and (prematurely) end of study report 28

10.6 Public disclosure and publications policy 28

11. STRUCTURED RISK ANALYSIS 30

11.1 Potential issues of concern 30

11.2 Synthesis 30

12. REFERENCES 31

# LIST OF ABBREVIATIONS AND RELEVANT DEFINITIONS

| ABR | ABR form, General Assessment and Registration form, is the application form that is required for submission to the accredited Ethics Committee (In Dutch, ABR = Algemene Beoordeling en Registratie) |
| --- | --- |
| AE | Adverse Event |
| AVG | General Data Protection Regulation (in Dutch Algemene Verordering Persoonsgegevens) |
| AHSP | Appetite, Hunger and Sensory Perception |
| AR | Adverse Reaction |
| CCMO | Central Committee on Research Involving Human Subjects; in Dutch: Centrale Commissie Mensgebonden Onderzoek |
| CTN | Chorda tympani nerve |
| CV | Curriculum Vitae |
| DSMB | Data Safety Monitoring Board |
| eCRF | electronic Case Report Form |
| EGM | Electrogustometer |
| GCP | Good Clinical Practice |
| IB | Investigator’s Brochure |
| IC | Informed Consent |
| METC | Medical research ethics committee (MREC); in Dutch: medisch ethische toetsing commissie (METC) |
| MTPRT | Macronutrient and Taste Preference Ranking Task |
| (S)AE | (Serious) Adverse Event |
| SPC | Summary of Product Characteristics (in Dutch: officiële productinfomatie IB1-tekst) |
| Sponsor | The sponsor is the party that commissions the organisation or performance of the research, for example a pharmaceutical  company, academic hospital, scientific organisation or investigator. A party that provides funding for a study but does not commission it is not regarded as the sponsor, but referred to as a subsidising party. |
| SUSAR | Suspected Unexpected Serious Adverse Reaction |
| TST | Taste Strip Test |
| WMO | Medical Research Involving Human Subjects Act (in Dutch: Wet Medisch-wetenschappelijk Onderzoek met Mensen) |

**SUMMARY**

**Rationale:** The chorda tympani nerve (CTN) is a mixed nerve, which carries sensory and parasympathetic fibers. The sensory component supplies the taste sensation of the anterior two-thirds of the ipsilateral side of the tongue. During middle ear surgery the CTN is exposed and frequently stretched or sacrificed, because it lacks a bony covering as it passes the middle ear. The injury may cause hypogeusia, ageusia or altered taste sensation of the ipsilateral side of the tongue. Patients may also suffer from a dry mouth. To date, there is no consensus regarding which type of CTN injury, obtained during primary stapes surgery and primary cochlear implantation, gives the least burden.

**Objective:** To determine whether damaging the CTN during primary stapes surgery and primary cochlear implantation influences postoperative taste function and to determine which type of injury is associated with worse postoperative taste function. Taste function will be measured using taste strips.

**Study design:** A prospective study.

**Study population:** Adult patients, 18 years and older, who are eligible for primary stapes surgery or primary cochlear implantation

**Main study parameter:** Amount of identified taste strips of the ipsilateral tongue to the operated ear (range 0 – 20).

**Nature and extent of the burden and risks associated with participation, benefit and group relatedness:** Patients will not experience any risks during participation in the study. There will be four test sessions. Each time, all participants complete two taste tests and a macronutrient an taste preference ranking task and fill out several sets of questionnaires. The test sessions will take place in the University Medical Center Utrecht during the regular visits of the patient to the hospital. Three months after surgery the taste sensation is evaluated at home using questionnaires. The study burn is moderate. We believe that the burden is in proportion to the potential value of the research.

# 1. INTRODUCTION AND RATIONALE

The chorda tympani nerve (CTN), a branch of the facial nerve, is a mixed nerve, which carries sensory and parasympathetic fibers. The sensory component supplies the taste sensation of the anterior two-thirds of the ipsilateral side of the tongue. The parasympathetic component innervates the submandibular and sublingual salivary gland. The CTN passes the medial surface of the neck of the malleus and runs in between the malleus and the incus in the tympanic cavity. The CTN leaves the tympanic cavity together with the anterior tympanic artery through the anterior canaliculus. The nerve continues through the petrotympanic fissure and runs towards the infratemporal fossa. This is where the CTN merges with the lingual nerve. During middle ear surgery, the CTN is exposed and frequently stretched or sacrificed, because it lacks a bony covering as it passes the middle ear. The injury may cause hypogeusia, ageusia or altered taste sensation of the ipsilateral side of the tongue. Patients may also suffer from a dry mouth (1).

To date, there is no consensus regarding which type of CTN injury, obtained during primary stapes surgery or primary cochlear implantation, gives the least burden. Some articles concluded that the taste outcome is better if the CTN is sacrificed instead of the CTN being preserved but damaged by stretching (2–5). Other articles report that sacrificing the CTN results in more taste disturbance compared to nerve preservation (6–9). Lastly, Rice et al. concluded that sacrificing or preserving the CTN makes little difference for the patient. Sparing the CTN may hinder an adequate view of the middle ear structures during surgery. If in fact sacrificing the CTN leads to less or equal taste disturbance compared to stretching it, sparing the CTN would not be justified (10).

A recently published systematic review of the effect of CTN injury during noninflammatory middle ear surgery concluded that patients with a stretched CTN were slightly more symptomatic than patients with a sacrificed CTN. The included studies were characterized by several limitations in their study design. The follow-up duration varied greatly between the included studies with a range of 6 weeks to 99 months. A longer follow-up duration may result in fewer symptoms due to the longer recovery time or habituation of symptoms (1). Secondly, age seems to be associated with recovery rate. Younger patients seem to have significantly higher recovery rates of CTN function compared to older patients (11). Most of the included studies disregarded age. Thirdly, postoperative taste dysfunction may have been underreported. It is not clear whether surgeon routinely asked their patients about taste dysfunction in the included studies. Lastly, most studies did not include quality of life as an outcome measure and therefore it remains unclear whether taste dysfunction significantly impacts quality of life (1).

*Purpose of this study*

A lack of high quality studies on the subject precludes firm evidence-based recommendations and demonstrates the need for a high-quality study. In order to accommodate this need, in the proposed double-blind prospective study we will investigate the effect of CTN injury on postoperative taste disturbance and quality of life.

# 2. OBJECTIVES

Primary Objective:

To evaluate the effect of CTN injury on postoperative taste function measured using taste strips in patients undergoing primary stapes surgery or primary cochlear implantation.

Secondary Objective(s):

- To compare the postoperative taste function of sweet, sour, bitter, salty and umami measured using taste strips in patients between two types of CTN injury (stretching or sacrificing)
- To compare the postoperative EGM threshold in uA for different locations of the tongue in patients between two types of CTN injury (stretching or sacrificing)
- To compare the presence of postoperative symptoms of taste disturbance in patients between two types of CTN injury (stretching or sacrificing).
- To compare the postoperative perception of appetite, hunger and sensory in patients between two types of CTN injury (stretching or sacrificing).
- To compare the postoperative quality of life in patients between two types of CTN injury (stretching or sacrificing).
- To compare the postoperative enjoyment of food in patients between two types of CTN injury (stretching or sacrificing).
- To compare the postoperative food preference in patients between two types of CTN injury (stretching or sacrificing).
- To compare the postoperative odour identification score in patients between two types of CTN injury (stretching or sacrificing)

Other parameters:

- Age
- Gender
- Medical history
- Details of the stapes surgery or cochlear implantation

# 3. STUDY DESIGN

This study concerns a prospective study including patients who will undergo primary stapes surgery or primary cochlear implantation. The study will be performed at the department of Otorhinolaryngology in the UMCU.

The objective of this study is to evaluate the effect of CTN injury on postoperative taste function. CTN injury is a categorical variable with three categories: no touch, mobilisation and completely sectioned.

The study design is illustrated in Figure 1. Patients participate after Informed Consent (IC) is obtained. It will run for approximately two years in the UMCU. Evaluation will take place preoperatively and at 1 week, 6 weeks and 6 months postoperatively by means of taste strip test (TST), electrogustometer (EGM), Macronutrient and Taste Preference Ranking Task (MTPRT) and questionnaires (AHSP questionnaire, Health related quality of life questionnaire and a supplementary questionnaire on taste perception). Preoperatively and 1 week postoperatively the patient’s olfactory function will be evaluated using the Sniffin’ Sticks test. Three months after surgery the taste sensation is evaluated at home using questionnaires. The questionnaires will be sent by Castor or on paper. The contents of these evaluations will be explained in detail in the ‘Investigational products’ and ‘Methods’ section of this protocol.


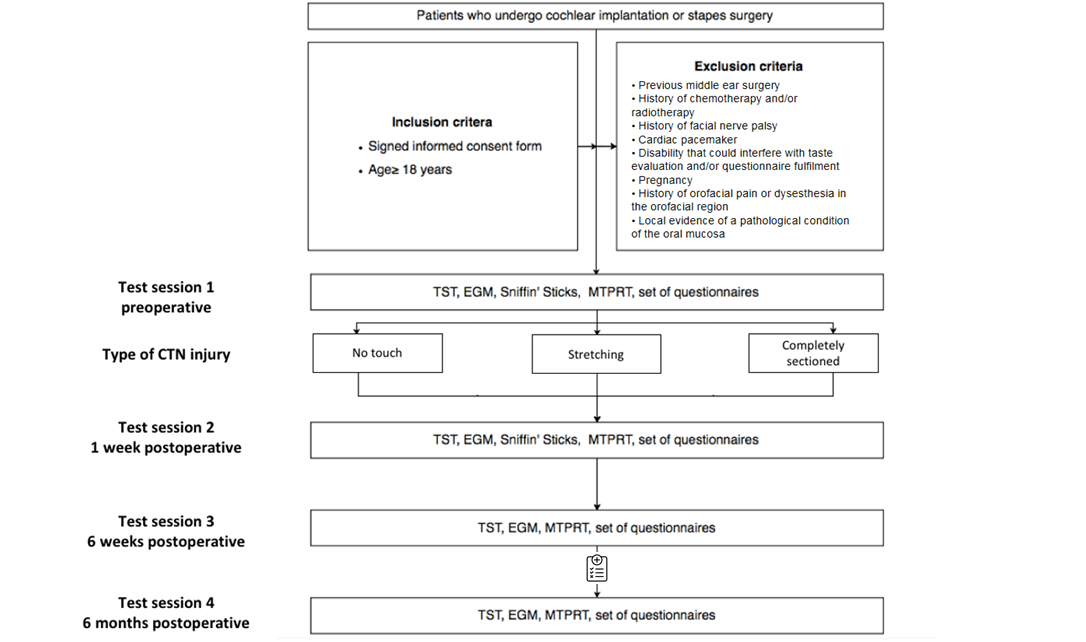


**Figure 1.** Flowchart study procedures. TST: taste strip test, EGM: electrogustometer, AHSP: appetite, hunger and sensory perception, MTPRT: macronutrient and taste preference ranking task The symbol
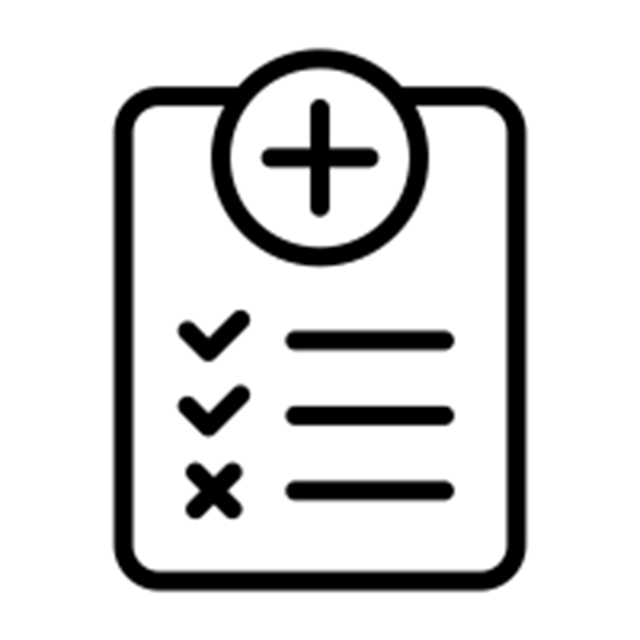
 means that three months after surgery the taste sensation will be evaluated at home using three questionnaires.

# 4. STUDY POPULATION

## 4.1 Population base

The study population consists of 154 adult patients who will undergo primary stapes surgery or primary cochlear implantation.

## 4.2 Inclusion criteria

In order to be eligible to participate in this study, a subject must meet all of the following criteria:

- Signed IC form
- Age ≥ 18 years
- Patients who are planned to undergo primary stapes surgery or primary cochlear implantation
- Willingness and ability to participate in all scheduled procedures outlined in the research protocol
- Good understanding of the Dutch language

## 4.3 Exclusion criteria

A potential subject who meets any of the following criteria will be excluded from participation in this study:

- Previous middle ear surgery (with the exception of the placement of ventilation tubes in childhood)
- History of chemotherapy and/or radiotherapy
- History of facial nerve palsy
- Cardiac pacemaker
- Disability that could interfere with taste evaluation and/or questionnaire fulfilment
- Pregnancy
- History of orofacial pain
- History of dysesthesia in the orofacial region
- Local evidence of a pathological condition of the oral mucosa

## 4.4 Sample size calculation

We will perform linear regression analysis for our primary outcome measure. Including our potentially confounding covariates, a total of 12 variables will be included in the model. The variables will be injury of the chorda tympani nerve, age, gender, smoking, weight, gastroesophageal reflux, olfactory dysfunction, salivary hypofunction, medication, rhinosinusitis/allergic rhinitis, previous COVID-19 and diabetes mellitus (12–22). We have calculated that these variables are 14 degrees of freedom in total. We will include 10 patients per degree of freedom in our linear regression model. To anticipate withdrawal of 10% of participants, we will include an extra 14 patients which means we will need a sample size of 154 patients.

At the ENT department at the UMCU, 80 otosclerosis patients undergo primary stapes surgery per year and 100 patients undergo cochlear implantation per year. Assuming a participation rate of 70%, we expect to able to include 154 patients in 15 months’ time.

# 5. NON-INVESTIGATIONAL PRODUCT 5.1 Taste Strip Test

We will also use the TST to investigate the taste ability. This objective taste test is validated in a population of healthy individuals by Mueller et al. The test will be performed according to their technique (30). The researcher will use filter paper strips, which are impregnated with four concentrations of sweet (0.4, 0.2, 0.1, and 0.05 g/mL sucrose), sour (0.3, 0.165, 0.09, and 0.05 g/mL citric acid), salty (0.25, 0.1, 0.04, and 0.016 g/mL sodium chloride), bitter (0,006, 0.0024, 0.0009, and 0.0004 g/mL quinine hydrochloride) and umami (0.03, 0.06, 0.12 and 0.25 g/mL sodium L glutamate monohydrate) tastes. Two blanked taste strips will also be used. We will present the strips in increasing concentrations in a randomized order to each side of the anterior tongue. The patient has to identify which one of the five flavours the strip is. The patient has to keep the tongue out of the mouth with the taste strip on the tongue, until he/she has made his decision by pointing to one of the five descriptors (sweet, sour, salty, bitter or umami) of the list. We will use 42 taste strips, because we want to test the taste ability of both sides of the tongue (30). Between the assessments of the taste strip the mouth will be rinsed with water. With this information we can compare the gustatory function of the area of the tongue that is innervated by the potentially injured CTN with the gustatory function of the contralateral area of the tongue. The patient will undergo this test preoperatively, 1 week, 6 weeks and 6 months postoperatively. Patients will be asked not to eat, drink (except for water), smoke or brush their teeth one hour before beginning the taste test (30). The taste strips will be ordered from Burghart Messtechnik (Germany).

**5.2 Sniffin’ Sticks**

We will use the Sniffin’ Sticks to test the olfactory function. This test is part of regular clinical practice at the UMCU and is validated in healthy Dutch adults (31). The Sniffin’ Sticks are pen-like devices, which are filled with liquids containing different odours. We will use 12 different odours. For testing, the researcher will firstly remove the cap of the pen-like device. Secondly the researcher will place the pens tip 2 cm in front of the nostrils for 3 seconds. After 3 seconds the patient has to identify the odour. The interval between the presentations of the different odours will be at least 30 seconds to prevent olfactory desensitization (32). The patient will undergo this test preoperatively and 1 week postoperatively. With this information it will be possible to conclude whether the olfactory function is intact or not. We will test the olfactory function, because the perception of flavour is generated by taste and smell sensation (33). Some studies concluded that smell sensation plays a more dominant role in the tasting of food than taste function (34). The Sniffin’ Sticks will also be ordered from Burghart Messtechnik (Germany).

**5.3 Macronutrient and Taste Preference Ranking Task**

We will use the MTPRT to test the food preference in patients. The test consists of pictures of products from four macronutrient categories. These four macronutrient categories are high-carbohydrate, high-fat, high-protein and low-energy. The MTPRT consists of three parts: practicing, liking and ranking. During the practicing part four combinations of four pictures are presented and patients will be asked to rank these pictures according to ‘what they most desire to eat at that moment’. During the liking part 32 pictures of products are presented with the question ‘How much do you like [product name]?’. Patients will be asked to rate this question on a 100-point visual analogue scale anchored by ‘do not like at all’ and ‘like extremely’. During the ranking part patients will be asked to rank according to ‘what they most desire to eat at that moment’. Participant will first click on the product they most desire to eat at the moment of completing the task. Afterwards they will click on the second most desired product, followed by the third most desired product and the product they least desired to eat at the moment of completing the task. Patients will be asked to refrain from eating and to drink only water or tea without sugar during the three hours prior to the test session (35). The patient will undergo this test preoperatively, 1 week, 6 weeks postoperatively and 6 months postoperatively.

# 5.4 Electrogustometer

We will use the CE marked EGM to investigate the taste ability. Different studies have established that the EGM is a good clinical tool for measuring taste detection thresholds (23–25). Electric taste, tastes like metal and sour, will be assessed with the Electrogustometer SI-03 (Sensonics Inc., Haddon Heights, NJ). We will detect the electric taste threshold with anodal stimulation. We will stimulate from 0.25 to 450 uA using the forced-choice single staircase test procedure. The EGM manual describes the two-down one-up forced-choice single staircase procedure (see appendix 1). This taste threshold setting procedure has a correct response level of 70.7% (26,27). The stimulus duration will be set at 0.5 sec. The lowest number of uA that generates an electric taste will be determined to be the value of the taste threshold of the CTN. We will measure the taste detection threshold on the anterior and lateral region of both sides of the tongue, so we can compare the taste thresholds of the ipsilateral side of the tongue to the operated ear with the contralateral side of the tongue (see figure 1). The patient will undergo this test preoperatively, 1 week, 6 weeks and 6 months postoperatively.


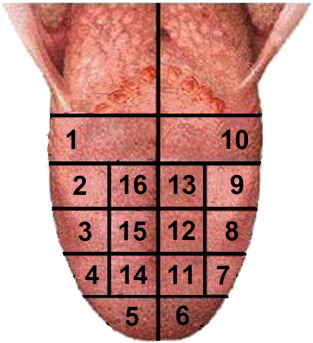


Figure 2. Anterior tongue regions: anterior tongue (regions 5 and 6), lateral tongue (regions 2,3,4,7,8 and 9), central tongue (regions 11,12,13,14,15 and 16) and posterior tongue (regions 1 and 10) (28).

Different studies have established that EGM is a good clinical tool for measuring taste detection thresholds (23–25). The EGM can also assess regional taste thresholds. Sensitivity of detection is lower on the ipsilateral side of the tongue to CTN transection compared to the contralateral side of the tongue. We will measure the taste threshold of the lateral sides of the tongue (see figure 2). (29). Stimulus duration affects the threshold values and therefore the stimulus duration will be set at 0.5 sec. for all participants (28).

## No potential risks are described in the literature. The EGM doesn’t connect to any outside voltage source and uses a series of AA batteries to produce it’s stimuli. In addition, the amount of current is confined to very low uA levels (0.25-450 uA) that are detected primarily by taste afferents. By using a dual electrode, no current flows through the body. Patients with a pacemaker could therefore be included in the study, but as a precaution we will exclude these patients. The EGM manual describes the precautions for proper use of the EGM (see appendix 1).

# 6. METHODS

## 6.1 Study parameters/endpoints

### 6.1.1 Main study parameter

The main study parameter will be the 6 weeks postoperative amount of identified taste strips of the ipsilateral tongue of the operated ear. The range for possible scores is 0 to 20

### 6.1.2 Secondary study parameters

- The difference between the preoperative and postoperative EGM thresholds in uA for different locations of the tongue
- The difference between the preoperative and postoperative amount of identified sweet taste strips of the ipsilateral tongue of the operated ear
- The difference between the preoperative and postoperative amount of identified salty taste strips of the ipsilateral tongue of the operated ear
- The difference between the preoperative and postoperative amount of identified sour taste strips of the ipsilateral tongue of the operated ear
- The difference between the preoperative and postoperative amount of identified bitter taste strips of the ipsilateral tongue of the operated ear
- The difference between the preoperative and postoperative amount of identified umami taste strips of the ipsilateral tongue of the operated ear
- The postoperative existence of metallic taste sensation
- The postoperative existence of dryness of the mouth
- The postoperative existence of tingling sensations of the tongue
- The postoperative existence of numbness of the tongue
- The difference between the preoperative and postoperative score of the AHSP questionnaire
- The difference between the preoperative and postoperative score of the health-related quality of life questionnaire
- The difference between the preoperative and postoperative total and sub scores of the MTPRT
- The difference between the preoperative and postoperative odour identification score

### 6.1.3 Other study parameters

Baseline characteristics (age, gender), medical history and details concerning the surgical procedure that was performed.

## 6.2 Randomisation, blinding and treatment allocation

There will be no randomization. The patient and outcome assessor are blinded to the predictor variables, including the presence or absence of CTN injury. The surgeon will complete an electronic Case Report Form (eCRF) in Castor, including the predictor variable CTNinjury. Both patients and researchers will not be informed about the CTN injury until the patient has finished all evaluations. The eCRF will be locked to read for the researchers and the surgeon will not report the CTN injury in the surgery report. After a patient has completed all evaluations, the investigator will read the eCRF and report the CTN injury in the electronic patient record. We do not expect that there are circumstances that require us to unblind the patient or the outcome assessor during the study.

## 6.3 Study procedures

The procedures are schematically presented in figure 1. For four times, all participants complete the two taste tests and the MTPRT and fill out the AHSP questionnaire, health related quality of life questionnaire and the supplementary questionnaire. Preoperative the tests and the fulfilment of the questionnaires take place once**.** After the middle ear surgery, the test session will repeat three times; after 1 week, 6 weeks and 6 months. During the preoperative and 1 week postoperative test session also the sniffin’ stick test will take place. Three months after surgery the taste sensation is evaluated at home using the three questionnaires. The other four test sessions will take place during the regular visits of the patient to the hospital. The surgeon will fill out an eCRF about the surgery. The eCRF will be sent by Castor.

*Explanation of the different questionnaires*

- eCRF: 5 multiple choice questions about the middle ear surgery.
- AHSP questionnaire: 33 questions answered on a 5 point Likert scale to assess self-judgement of taste and smell perception. Translated to Dutch and validated in 2001 (36).
- Health related quality of life questionnaire: 24 statements or questions about quality of life ranked by one of the following 6 options: agree, agree partly, disagree partly, disagree, I think this question has no value, I don’t understand the question. 7 questions about depression and anxiety require a yes or no answer. 4 questions answered on a 10 point Likert scale to assess impact of loss of taste function on daily life. This questionnaire is based on the questionnaire of olfactory disorder (37–39).
- Supplementary questionnaire: 5 questions to assess taste perception on a 10 point Likert scale and 10 questions about possible factors that may influence taste sensation.

## 6.4 Withdrawal of individual subjects

Subjects can leave the study at any time for any reason if they wish to do so without any consequences. The investigator can decide to withdraw a subject from the study for urgent medical reasons.

### 6.4.1 Specific criteria for withdrawal

Not applicable.

## 6.5 Replacement of individual subjects after withdrawal

Subjects who withdraw from the study or who terminate the recording session prematurely will be considered as lost to follow-up. To anticipate a withdrawal of 10% of patients, fourteen more patients than needed will be recruited. Therefore, there will be no reason to replace individual subjects after withdrawal, unless more than 10% of participants will withdraw.

## 6.6 Follow-up of subjects withdrawn from treatment

In case of withdrawal, the subject will remain in care of their own otorhinolaryngologist and continue with standard medical treatment.

## 6.7 Premature termination of the study

Serious adverse events (SAE) are not expected, but in case any SAE do occur, each member of the research group has the right to terminate the study prematurely. See also paragraph 8.2.2 Serious adverse events (SAE’s).

# 7. SAFETY REPORTING

## 7.1 Temporary halt for reasons of subject safety

In accordance to section 10, subsection 4, of the WMO, the sponsor will suspend the study if there is sufficient ground that continuation of the study will jeopardise subject health or safety. The sponsor will notify the accredited METC without undue delay of a temporary halt including the reason for such an action. The study will be suspended pending a further positive decision by the accredited METC. The investigator will take care that all subjects are kept informed.

## 7.2 AEs, SAEs and SUSARs

### 7.2.1 Adverse events (AEs)

Adverse events are defined as any undesirable experience occurring to a subject during the study, whether or not considered related to the use of the EGM or TST. All adverse events reported spontaneously by the subject or observed by the investigator or his staff will be recorded in Castor.

### 7.2.2 Serious adverse events (SAEs)

A serious adverse event is any untoward medical occurrence or effect that

- results in death’
- is life threatening (at the time of the event);
- requires hospitalisation or prolongation of existing inpatients’ hospitalisation;
- results in persistent or significant disability or incapacity
- is a congenital anomaly or birth defect; or
- any other important medical event that did not result in any of the outcomes listed above due to medical or surgical intervention but could have been based upon appropriate judgement by the investigator.

An elective hospital admission will not be considered as a serious adverse event.

None of the SAEs are expected as a result of our study. In case, for any unforeseen reason, if the SAE could be due to our study protocol, the study would be paused. An independent team of investigators will be asked to investigate the SAE. In case the independent team of investigators would conclude the SAE would be due to our study protocol, the study will be terminated immediately.

The investigator will report all SAEs to the sponsor without undue delay after obtaining knowledge of the events. The sponsor will report the SAEs through the web portal ToetsingOnline to the accredited METC that approved the protocol, within 7 days of first knowledge for SAEs that result in death or are life threatening followed by a period of maximum of 8 days to complete the initial preliminary report. All other SAEs will be reported within a period of maximum 15 days after the sponsor has first knowledge of the serious adverse events.

7.2.3 Suspected unexpected serious adverse reactions (SUSARs)
Not applicable.

## 7.3 Annual safety report

Not applicable.

## 7.4 Follow-up of adverse events

All AEs will be followed until they have abated, or until a stable situation has been reached. Depending on the event, follow up may require additional tests or medical procedures as indicated, and/or referral to the general physician or a medical specialist. SAEs need to be reported till end of study within the Netherlands, as defined in the protocol.

7.5 [Data Safety Monitoring Board (DSMB) / Safety Committee]
Based on the document Data and Safety Monitoring Board (Groenestyn M.A.C. et. Al, UMCU 7-38) a DSMB is not indicated.

# 8. STATISTICAL ANALYSIS

## All statistical analyses will be performed using the IBM SPSS Statistics version 21.0 software package (Chicago, Illinois, USA). A test for normality and histograms will be used to assess whether variables are normally distributed. We expect limited missing data. Potentially missing variables will be handled with multiple imputation.

## 8.1 Primary study parameter

The primary study parameter is the change in the amount of identified taste strips by patient, preoperatively and 6-8 weeks postoperatively. This outcome will be presented continuous with a range from 1 to 20 identified taste strips. Mean (or the median) and standard deviation (or the range) will be reported. The effect of CTN injury on the amount of identified taste strips will be evaluated using linear regression analysis. The following confounding variables will be included in the analysis: age, gender, smoking, weight, gastroesophageal reflux, olfactory dysfunction, salivary hypofunction, medication, rhinosinusitis/allergic rhinitis, previous COVID-19 and diabetes mellitus (12–22).

## 8.2 Secondary study parameters

EGM thresholds and the MTPRT will be reported preoperatively and 1 week, 6 weeks and 6 months postoperatively. The total score and scores of different categories of the questionnaires will be reported preoperatively and 1 week, 6 weeks, 3 months and 6 months postoperatively. The odour identification score will be reported preoperatively and 1 week postoperatively. Mean (or median) and standard deviation (or the range) will be reported. Differences between the different groups of CTN injury will be analysed using linear regression analysis.

Postoperative symptoms of taste disturbance will be analysed using logistic regression analysis, as these outcomes will be presented dichotomously.

## 8.3 Other study parameters

Baseline characteristics will be presented in medians and averages.

## 8.4 Interim analysis

No interim analyses will be performed.

# 9. ETHICAL CONSIDERATIONS

## 9.1 Regulation statement

The study will be conducted according to the principles of the Declaration of Helsinki (WMA Declaration of Helsinki – Ethical Principles for Medical Research Involving Human Subjects. 64^th^ WMA General Assembly, Fortaleza, Brazil, October 2013) and in accordance with the Medical Research Involving Human Subjects Act (WMO, the ‘gedragscode gezondheidsonderzoek’.

## 9.2 Recruitment and consent

Patients will be recruited from the outpatient ENT department of the UMCU. Patients will be asked to participate in the study after the decision has been made that non inflammatory surgery of the middle ear, like cochlear implant surgery or surgery of the ossicles is needed. The treating physician will provide the patient with the information letter and informed consent form and will ask the patient for permission to be contacted by the research team. Patients will be contacted by the researcher after at least one week to answer potential questions. The next visit to the UMCU the patient can bring the IC form where it can be signed together with the investigator. In most cases, the IC form will be signed after the preoperative visit to the treating physician. The treating physician is not present when signing the IC form. The patient will receive a copy of the signed IC. The informed consent will also documented in Hix. If the patient will sign the IC form, the patient will receive the first test session at the same day. If a patient does not want to participate, contact with the investigator will be terminated.

## 9.3 Objection by minors or incapacitated subjects

If a patient does not want to receive the EGM or the TST, the patient will not be forced. The patient will be excluded from the study. Only competent patients 18 years of age or older will be included in our study.

## 9.4 Benefits and risks assessment, group relatedness

Patients will not have a direct benefit of this study, but with these results of the study there will be more scientific knowledge about de postoperative taste function after primary stapes surgery or primary cochlear implantation.

The study burden is moderate. All patients will receive the tests, in the context of the study, during the regular visits to the Department of Otorhinolaryngology. The combination of the fulfilment of the questionnaires and receiving the EGM, the TST and the MTPRT the olfactory test with Sniffin’ sticks will take 1.5 hour. All patients will undergo these tests for 4 times. Except for the sniffin stick test, this will only be done twice. Patient will also complete the questionnaires at home three months after surgery, which will take 30 minutes. The expected total burden due to participation for all patients is likely to be 6.5 hours.

The EGM, TST and the olfactory test are considered as non-risk investigations.

## 9.5 Compensation for injury

The sponsor/investigator has liability insurance which is in accordance with article 7 of the WMO.

The sponsor (also) has an insurance which is in accordance with the legal requirements in the Netherlands (article 7 WMO). This insurance provides cover for damage to research subjects through injury or death caused by the study. The insurance applies to the damage that becomes apparent during the study or within 4 years after the end of the study.

## 9.6 Incentives

Not applicable.

# 10. ADMINISTRATIVE ASPECTS, MONITORING AND PUBLICATION

## 10.1 Handling and storage of data and documents

Data handling and protection is conducted according to the ISO 27001 compliant processes and ICH-GCP and applicable regulations. Confidentiality will be maintained at all times, participant information will not be disclosed to third parties. For this prospective study, only eligible patients admitted or with an appointment at the ENT department will be asked by their treating physician to participate. Direct identifiable—personal data from these patients will be recorded in an Excel file and stored in a secure research folder from the TACO study on the UMC Utrecht network drive. This is for an overview of which patients are asked to participate.

After given informed consent, patients will also be registered in our electronic health record (EHR, HiX). The original signed informed consent forms will be kept in a binder in a locked closet in a locked room at the ENT department. Subsequently, the patient will receive a unique identifier, after which members of the research team will extract all necessary clinical parameters from the electronic health records (EHRs; HiX) into an electronic Case Report Form (eCRF) the UMCU endorsed system Castor EDC. Castor EDC is a browser-based, metadata-driven EDC software solution and workflow methodology for building and managing online databases. The eCRF contains data items as specified in this research protocol. Modification of the eCRF will be made only if deemed necessary and in accordance with an amendment to the research protocol. Access to the eCRF is password protected and specific roles are assigned (e.g. study coordinator, investigator, monitor, etc.).

Only research members directly involved in this study will get access to the all of the collected in Castor. The clinical database will be supplemented with the outcome of three questionnaires (please see paragraph 7.3 Study Procedures). At the end of the study, all generated (meta)data will be stored as a proprietary format (.sav / .xlsx format) in a secure Research Folder Structure with access control. Only members of the research team who are directly involved in the study are allowed to access the key-linking table to enable patient re-identification (only for UMCU patients). In order to reproduce the study findings and to help future users to understand and reuse the (meta)data, all changes made to the raw data, including analysis steps will be documented in an analysis plan. Thus the secure research folder will serve at the end of the study as a data package. The data package will be locally archived on the secured research network disc of the division for 15 years after the study has ended. More details, including the state of FAIRness, can be found in our datamanagement plan on [https://dmponline.dcc.ac.uk/plans/71095](https://dmponline.dcc.ac.uk/plans/__.).

## 10.2 Monitoring and Quality Assurance

Our datamanagement plan can be found on [https://dmponline.dcc.ac.uk/plans/71095](https://dmponline.dcc.ac.uk/plans/__.). See the attached monitor plan.

## 10.3 Amendments

Amendments are changes made to the research after a favourable opinion by the accredited METC has been given. All amendments will be notified to the METC that gave a favourable opinion.

## 10.4 Annual progress report

The sponsor/investigator will submit a summary of the progress of the study to the accredited METC once a year. Information will be provided: the date of inclusion of the first subject, numbers of subjects included and numbers of subjects that have completed the study, serious adverse events/serious adverse reactions, other problems, and amendments.

## 10.5 Temporary halt and (prematurely) end of study report

The investigator will notify the accredited METC of the end of the study within a period of 8 weeks. The end of the study is defined as the last patient’s last visit to the UMCU.

The sponsor will notify the METC immediately of a temporary halt of the study, including the reason of such an action.

In case the study is ended prematurely, the sponsor will notify the accredited METC within 15 days, including the reasons for the premature termination.

Within one year after the end of the study, the investigator/sponsor will submit a final sturdy report with the results of the study, including any publications/abstracts of the study, to the accredited METC.

## 10.6 Public disclosure and publications policy

The data of this research will be used for publication in peer-reviewed international journals. Both positive and negative study results will be disclosed. If the journals do not consider our results for publication, the research will be disclosed through study registers, websites or databases.

# 11. STRUCTURED RISK ANALYSIS

## 11.1 Potential issues of concern

Not applicable.

## 11.2 Synthesis

## No potential risks are described in the literature. The EGM is CE marked and will be used within de indication. The EGM doesn’t connect to any outside voltage source and uses a series of AA batteries to produce it’s stimuli. In addition, the amount of current is confined to very low uA levels that are detected primarily by taste afferents. By using a dual electrode, no current flows through the body. Patients with a pacemaker could therefore be included in the study, but as a precaution we will exclude these patients. The EGM manual describes the precautions for proper use of the EGM (see appendix 1).

# 12. REFERENCES

1. Ziylan F, Smeeing DPJ, Bezdjian A, Stegeman I, Thomeer HGXM. Feasibility of preservation of chorda tympani nerve during noninflammatory ear surgery: A systematic review. Laryngoscope. 2018 Aug;128(8):1904–13.

2. Lloyd S, Meerton L, Di Cuffa R, Lavy J, Graham J. Taste change following cochlear implantation. Cochlear Implants Int. 2007 Dec;8(4):203–10.

3. Clark MPA, O’Malley S. Chorda tympani nerve function after middle ear surgery. Otol Neurotol Off Publ Am Otol Soc Am Neurotol Soc [and] Eur Acad Otol Neurotol. 2007 Apr;28(3):335–40.

4. Gopalan P, Kumar M, Gupta D, Phillipps JJ. A study of chorda tympani nerve injury and related symptoms following middle-ear surgery. J Laryngol Otol. 2005 Mar;119(3):189–92.

5. Michael P, Raut V. Chorda tympani injury: operative findings and postoperative symptoms. Otolaryngol neck Surg Off J Am Acad Otolaryngol Neck Surg. 2007 Jun;136(6):978–81.

6. Yeo SB, Loy AH. Chorda tympani trauma--how much does it affect taste? Singapore Med J. 1997 Aug;38(8):329–31.

7. Mahendran S, Hogg R, Robinson JM. To divide or manipulate the chorda tympani in stapedotomy. Eur Arch oto-rhino-laryngology Off J Eur Fed Oto-Rhino-Laryngological Soc Affil with Ger Soc Oto-Rhino-Laryngology - Head Neck Surg. 2005 Jun;262(6):482–7.

8. Yung M, Smith P, Hausler R, Martin C, Offeciers E, Pytel J, et al. International Common Otology Database: taste disturbance after stapes surgery. Otol Neurotol Off Publ Am Otol Soc Am Neurotol Soc [and] Eur Acad Otol Neurotol. 2008 Aug;29(5):661–5.

9. Berling Holm K, Knutsson J, Strömbäck K, Danckwardt Lillieström N, Papatziamos G, Rosenblad A, et al. Taste disturbance after stapes surgery: an evaluation of frequency, severity, duration, and quality-of-life. Acta Otolaryngol. 2017 Jan;137(1):39–43.

10. Rice JC. The chorda tympani in stapedectomy. J Laryngol Otol. 1963 Nov;77:943–4.

11. Sone M, Sakagami M, Tsuji K, Mishiro Y. Younger patients have a higher rate of recovery of taste function after middle ear surgery. Arch Otolaryngol Head Neck Surg. 2001 Aug;127(8):967–9.

12. Le Floch JP, Lièvre G, Labroue M, Peynègre R, Perlemuter L. Early detection of diabetic patients at risk of developing degenerative complications using electric gustometry: a five-year follow-up study. Eur J Med. 1992 Jul 1;1:208–14.

13. Grant R, Ferguson MM, Strang R, Turner JW, Bone I. Evoked taste thresholds in a normal population and the application of electrogustometry to trigeminal nerve disease. J Neurol Neurosurg Psychiatry. 1987 Jan;50(1):12–21.

14. Kabadi A, Saadi M, Schey R, Parkman HP. Taste and Smell Disturbances in Patients with Gastroparesis and Gastroesophageal Reflux Disease. J Neurogastroenterol Motil. 2017 Jul;23(3):370–7.

15. Kinnaird E, Stewart C, Tchanturia K. Taste sensitivity in anorexia nervosa: A systematic review. Int J Eat Disord. 2018 Aug;51(8):771–84.

16. Muñoz-González C, Vandenberghe-Descamps M, Feron G, Canon F, Labouré H, Sulmont-Rossé C. Association between Salivary Hypofunction and Food Consumption in the Elderlies. A Systematic Literature Review. J Nutr Health Aging. 2018;22(3):407–19.

17. Nishihara T, Nozaki T, Sawamoto R, Komaki G, Miyata N, Hosoi M, et al. Effects of Weight Loss on Sweet Taste Preference and Palatability following Cognitive Behavioral Therapy for Women with Obesity. Obes Facts. 2019;12(5):529–42.

18. Schiffman SS, Gatlin CA. Clinical physiology of taste and smell. Annu Rev Nutr. 1993;13:405–36.

19. Schiffman SS. Influence of medications on taste and smell. World J Otorhinolaryngol - head neck Surg. 2018 Mar;4(1):84–91.

20. Skrandies W, Zschieschang R. Olfactory and gustatory functions and its relation to body weight. Physiol Behav. 2015 Apr;142:1–4.

21. Tong JY, Wong A, Zhu D, Fastenberg JH, Tham T. The Prevalence of Olfactory and Gustatory Dysfunction in COVID-19 Patients: A Systematic Review and Meta-analysis. Otolaryngol neck Surg Off J Am Acad Otolaryngol Neck Surg. 2020 Jul;163(1):3–11.

22. Veček NN, Mucalo L, Dragun R, Miličević T, Pribisalić A, Patarčić I, et al. The Association between Salt Taste Perception, Mediterranean Diet and Metabolic Syndrome: A Cross-Sectional Study. Nutrients. 2020 Apr;12(4).

23. Murphy C, Quiñonez C, Nordin S. Reliability and validity of electrogustometry and its application to young and elderly persons. Chem Senses. 1995 Oct;20(5):499–503.

24. Stillman JA, Morton RP, Hay KD, Ahmad Z, Goldsmith D. Electrogustometry: strengths, weaknesses, and clinical evidence of stimulus boundaries. Clin Otolaryngol Allied Sci. 2003 Oct;28(5):406–10.

25. Stillman JA, Morton RP, Goldsmith D. Automated electrogustometry: a new paradigm for the estimation of taste detection thresholds. Clin Otolaryngol Allied Sci. 2000 Apr;25(2):120–5.

26. Bagla R, Klasky B, Doty RL. Influence of stimulus duration on a regional measure of NaCl taste sensitivity. Chem Senses. 1997 Apr;22(2):171–5.

27. Wetherill GB, Levitt H. Sequential estimation of points on a psychometric function. Br J Math Stat Psychol. 1965 May;18:1–10.

28. Pavlidis P, Schittek G, Saratziotis A, Ferfeli M, Kekes G, Gouveris H. Topological electrogustometry and chemogustometry surrogate markers of age-related gustatory decline in humans [Internet]. Authorea, Inc.; Available from: https://doi.org/10.22541%2Fau.158740046.62821264

29. Doty RL, Heidt JM, MacGillivray MR, Dsouza M, Tracey EH, Mirza N, et al. Influences of age, tongue region, and chorda tympani nerve sectioning on signal detection measures of lingual taste sensitivity. Physiol Behav. 2016;155:202–7.

30. Mueller C, Kallert S, Renner B, Stiassny K, Temmel AFP, Hummel T, et al. Quantitative assessment of gustatory function in a clinical context using impregnated “taste strips”. Rhinology. 2003 Mar;41(1):2–6.

31. Boesveldt S, Verbaan D, Knol DL, van Hilten JJ, Berendse HW. Odour identification and discrimination in Dutch adults over 45 years. Rhinology. 2008 Jun;46(2):131–6.

32. Hummel T, Sekinger B, Wolf SR, Pauli E, Kobal G. “Sniffin” sticks’: olfactory performance assessed by the combined testing of odor identification, odor discrimination and olfactory threshold. Chem Senses. 1997 Feb;22(1):39–52.

33. Boltong A, Keast R, Aranda S. Experiences and consequences of altered taste, flavour and food hedonics during chemotherapy treatment. Support care cancer Off J Multinatl Assoc Support Care Cancer. 2012 Nov;20(11):2765–74.

34. Spence C. Just how much of what we taste derives from the sense of smell? Flavour. 2015 Dec 1;4.

35. de Bruijn SEM, de Vries YC, de Graaf C, Boesveldt S, Jager G. The reliability and validity of the Macronutrient and Taste Preference Ranking Task: A new method to measure food preferences. Food Qual Prefer. 2017;57:32–40.

36. Mathey MF, de Jong N, de Groot CPGM, de Graaf C, van Staveren WA. Assessing appetite in Dutch elderly with the Appetite, Hunger and Sensory Perception (AHSP) questionnaire. J Nutr Health Aging. 2001;5(1):22–8.

37. Frasnelli J, Hummel T. Olfactory dysfunction and daily life. Eur Arch oto-rhino-laryngology Off J Eur Fed Oto-Rhino-Laryngological Soc Affil with Ger Soc Oto-Rhino-Laryngology - Head Neck Surg. 2005 Mar;262(3):231–5.

38. Philpott CM, Boak D. The impact of olfactory disorders in the United kingdom. Chem Senses. 2014 Oct;39(8):711–8.

39. Langstaff L, Pradhan N, Clark A, Boak D, Salam M, Hummel T, et al. Validation of the olfactory disorders questionnaire for English-speaking patients with olfactory disorders. Clin Otolaryngol Off J ENT-UK ; Off J Netherlands Soc Oto-Rhino-Laryngology Cerv-fac Surg. 2019 Sep;44(5):715–28.
