# Supplementary material for: The impact of injury of the chorda tympani nerve during primary stapes surgery or cochlear implantation on taste function, quality of life and food preferences: A study protocol for a double-blind prospective prognostic association study
Source: PLoS One. 2023 May 18;18(5):e0284571. doi: 10.1371/journal.pone.0284571 (PMC10194866; doi:10.1371/journal.pone.0284571)
Supplement: S3 File — (PDF) [file pone.0284571.s003.pdf]

S3 Attachment D – Informed consent form subject

Postoperative taste function after chorda tympani injury

TACO study

- I have read the information letter. I could also ask questions. My questions have been answered well enough. I had enough time to decide whether to participate.

- I know that participation is voluntary. I also know that I can make a decision at any time not to participate in the study. Or to stop. I don't have to say why I want to stop.

- I give the researcher permission to inform my general practitioner and specialist who treats me that I am participating in this study.

- I give the researcher permission to request information from my specialist who treated me about the performed stapes surgery or cochlear implantation.

- I give the researcher permission to provide my general practitioner or specialist with information about interim conclusions from the study that are important for my health.

- I give the researcher permission to collect and use my data. The researchers only do this to answer the research question of this study.

- I know that some people can see all my personal data for the purpose of checking the research. Those people are listed in this information letter. I give these people permission to view my personal data for this check.

- I know that if I become pregnant during this study, I will no longer be able to participate in this study.

- Do you want to tick yes or no in the table below?

I give permission to keep my data to use it for other research projects, as stated in the information letter.	Yes ☐	No ☐
I give permission to be asked if I want to participate in a follow-up study after this study.	Yes ☐	No ☐
I give permission to the researchers to let me know after the study is done whether there was any damage to the chorda tympani during the surgery.	Yes ☐	No ☐
I give the researchers permission to inform me of the main outcomes of the study.	Yes ☐	No ☐

- I want to participate in this study.

My name is (subject):

Signature Date: __ / __ / __

Ik have stated that I thoroughly informed this subject about the aforementioned study.

Will information become known during the research that could influence the subject's consent? Then I will let this test subject know in time.

Name researcher (or his/her representative):

Signature Date: __ / __ / __

Additional information has been provided by:

Name:

Function:

Signature: Date: __ / __ / __

A signed copy of the informed consent form is given to the subject together with an information letter.
