# Supplementary material for: The impact of injury of the chorda tympani nerve during primary stapes surgery or cochlear implantation on taste function, quality of life and food preferences: A study protocol for a double-blind prospective prognostic association study
Source: PLoS One. 2023 May 18;18(5):e0284571. doi: 10.1371/journal.pone.0284571 (PMC10194866; doi:10.1371/journal.pone.0284571)
Supplement: Attachment — Submitted filename: Response to reviewers.pdf [file pone.0284571.s004.pdf]

Dear Dr. Maria Elisabeth Johanna Zalm,

Thank you for considering our manuscript. Enclosed you will find our answers to the questions.

Yours sincerely,

Esther E. Blijleven, Robert J. Stokroos, Inge Wegner and Hans G.X.M. Thomeer

Journal Requirements

1. Please ensure that your manuscript meets PLOS ONE's style requirements, including those for file naming. The PLOS ONE style templates can be found at https://journals.plos.org/plosone/s/file?id=wjVg/PLOSOne_formatting_sample_main_body.pdf and https://journals.plos.org/plosone/s/file?id=ba62/PLOSOne_formatting_sample_title_authors_affiliations.pdf	Thank you for your comments. We have changed the filenames and the manuscript conforms to the style requirements.
2. We note that you have indicated that data from this study are available upon request. PLOS only allows data to be available upon request if there are legal or ethical restrictions on sharing data publicly. For more information on unacceptable data access restrictions, please see http://journals.plos.org/plosone/s/data-availability#loc-unacceptable-data-access-restrictions.<br><br>a) If there are ethical or legal restrictions on sharing a de-identified data set, please explain them in detail (e.g., data contain potentially sensitive information, data are owned by a third-party organization, etc.) and who has imposed them (e.g., an ethics committee). Please also provide contact information for a data access committee, ethics committee, or other institutional body to which data requests may be sent.<br><br>b) If there are no restrictions, please upload the minimal anonymized data set necessary to replicate your study findings as either Supporting Information files or to a stable, public repository and provide us with the relevant URLs, DOIs, or accession	This manuscript is a study protocol. We cannot share any data because we do not have results yet. The intention is to submit the manuscript containing the results of this study to PLOS one. We will publish an anonymized data set of the results of our research at that time.

numbers. For a list of acceptable repositories, please see http://journals.plos.org/plosone/s/data-availability#loc-recommended-repositories. We will update your Data Availability statement on your behalf to reflect the information you provide.	
3. Your ethics statement should only appear in the Methods section of your manuscript. If your ethics statement is written in any section besides the Methods, please move it to the Methods section and delete it from any other section. Please ensure that your ethics statement is included in your manuscript, as the ethics statement entered into the online submission form will not be published alongside your manuscript.	We have moved the ethics statement from the declaration section to the methods section. (see page 15-16, line 379-388)
4. Please include captions for your Supporting Information files at the end of your manuscript, and update any in-text citations to match accordingly. Please see our Supporting Information guidelines for more information: http://journals.plos.org/plosone/s/supporting-information.	We have included a caption of the supporting information files at the end of our manuscript. (see page 20, line 496-499)

Reviewer 1

This is an interesting double-blind prognostic study, designed to investigate the effect of CTN injury on postoperative taste function, defined as the postoperative taste strip score, in patients undergoing primary stapes surgery or primary cochlear implantation.	Thank you for your comments.
1. This has been stated to be a prognostic study- can the authors be more explicit in terms of design , i.e whether this is a description, association, prediction and causation, from the stats methods, its	The study is a prognostic association study. **Addition manuscript:** Double-blind prospective prognostic association study (see page 1, line 4-5; page 3, line 54 and page 7, line 146)

suggestive of either description or association.	
2. And consider whether these guidelines need to be used; PROGRESS, TRIPOD and CHARMS	We will not make a prediction model with the results, so the PROGRESS, TRIPOD, CHARMS guidelines are not applicable to our study.
3. Pre-specified covariates have been stated to include 14 variables. In the statistical analysis section, it would make sense to include univariate analysis of these covariates.	We will also perform univariate analysis of these covariates. **<u>Addition manuscript:</u>** Univariable and multivariable analysis will be performed. (see page 15, line 360-361)
4. In addition state, are the authors going to consider multicollinearity?	We will consider (multi)collinearity by assessing the Variation Inflation Factor (VIF) for each independent variable in SPSS. **<u>Additon manuscript:</u>** Multicollinearity will be considered by assessing the Variation Inflation Factor (VIF) for each independent variable in SPSS. (see page 15, line 357-359)
5. Any consideration of moderators or mediating factors?	We will consider effect modification by introducing interaction terms into the multivariable model (predictor*potential effect modifier). **<u>Addition manuscript:</u>** effect modification will be considered by introducing interaction terms into the multivariable model (predictor*potential effect modifier). (see page 15, line 359-360)
6. In the statistical analysis section, it might also be useful to include that, if assumption of normality are violated for the continuous outcome i.e the primary outcome - non parametric methods will be used.	We will use non-parametric methods if assumption of normality is violated. **<u>Addition manuscript:</u>** Parametric methods will be used if data are normally distributed and non-parametric methods will be used if assumption of normality is violated. (see page 14, line 349-350)

Reviewer 2

The reviewer thanks for the opportunity to analyze this work. It is a good written protocol.	Thank you for your comments.
1. Converted all “we…” in passive form.	We have converted all text from active to passive. (see manuscript)
2. Ear malformation should be considered in the exclusion criteria	Ear malformation is an exclusion criteria. **<u>Addition manuscript:</u>** Exclusion criteria: ear malformation. (see page 8, line 175)
3. Better describe surgical procedure performed and the type of method	The surgical method will be microscopic for all surgeries.

(endoscopic or microscopic).<br>Read: (doi:10.1177/0194599821990669; DOI: 10.5152/iao.2017.3322; DOI: 10.7874/jao.2021.00388).	
4. Eventual other post-surgical complications should be collected.	We will collect all adverse events, including all post-surgical complications.
5. Why will you not perform some valuations at all follow-up times? for example, the olfactory function evaluation?	We will not perform the Sniffin' Sticks test at all follow-up times to limit the burden on patients. The Institutional Review Board of the UMCU closely monitors the burden of patients participating in research and therefore we must be critical of the tests to which we expose the patient. After consultation of the Institutional Review Board of the UMCU we have decided to only perform the Sniffin' Sticks test twice, because we expect the sense of smell does not change (much) over time.
